# Development of Epirubicin-Loaded Biocompatible Polymer PLA–PEG–PLA Nanoparticles: Synthesis, Characterization, Stability, and In Vitro Anticancerous Assessment

**DOI:** 10.3390/polym13081212

**Published:** 2021-04-09

**Authors:** Salam Massadeh, Iman Almohammed, Eman Barhoush, Mustafa Omer, Nouf Aldhawi, Abdulaziz Almalik, Manal Alaamery

**Affiliations:** 1 Developmental Medicine Department, King Abdullah International Medical Research Center, King Saud Bin Abdulaziz University for Health Sciences, King Abdulaziz Medical City, Ministry of National Guard- Health Affairs (MNG-HA), Riyadh 11481, Saudi Arabia; almohammed.iman@gmail.com (I.A.); eman_omar2@hotmail.com (E.B.); aldhawino@ngha.med.sa (N.A.); 2 KACST-BWH Centre of Excellence for Biomedicine, Joint Centers of Excellence Program, King Abdulaziz City for Science and Technology (KACST), Riyadh 11442, Saudi Arabia; aalmalik@kacst.edu.sa; 3 Saudi Human Genome Project (SHGP), King Abdulaziz City for Science and Technology (KACST), Satellite Lab at King Abdulaziz Medical City (KAMC), Ministry of National Guard Health Affairs (MNG-HA), Riyadh 11426, Saudi Arabia; 4 College of Pharmacy, King Saud bin Abdulaziz University for Health Sciences, Riyadh 11481, Saudi Arabia; Ahmedm@ksau-hs.edu.sa; 5 King Abdullah International Medical Research Center, Riyadh 11481, Saudi Arabia; 6 Life Sciences and Environment Research Institute, King Abdulaziz City for Science and Technology (KACST), Riyadh 11442, Saudi Arabia

**Keywords:** epirubicin, biocompatible, polymers, nanoparticles, stability studies, sustained release, double emulsion

## Abstract

Epirubicin (EPI) is an anti-cancerous chemotherapeutic drug that is an effective epimer of doxorubicin with less cardiotoxicity. Although EPI has fewer side effects than its analog, doxorubicin, this study aims to develop EPI nanoparticles as an improved formula of the conventional treatment of EPI in its free form. Methods: In this study, EPI-loaded polymeric nanoparticles (EPI-NPs) were prepared by the double emulsion method using a biocompatible poly (lactide) poly (ethylene glycol) poly(lactide) (PLA–PEG–PLA) polymer. The physicochemical properties of the EPI-NPs were determined by dynamic light scattering (DLS), transmission electron microscopy (TEM), differential scanning calorimetry (DSC), entrapment efficiency and stability studies. The effect of EPI-NPs on cancer cells was determined by high throughput imaging and flow cytometry. Results: The synthesis process resulted in monodisperse EPI-NPs with a size of 166.93 ± 1.40 nm and an elevated encapsulation efficiency (EE) of 88.3%. In addition, TEM images revealed the spherical uniformness of EPI-NPs with no aggregation, while the cellular studies presented the effect of EPI-NPs on MCF-7 cells’ viability; after 96 h of treatment, the MCF-7 cells presented considerable apoptotic activity. The stability study showed that the EPI-NPs remained stable at room temperature at physiological pH for over 30 days. Conclusion: EPI-NPs were successfully encapsulated within a highly stable biocompatible polymer with minimal loss of the drug. The used polymer has low cytotoxicity and EPI-NPs induced apoptosis in estrogen-positive cell line, making them a promising, safe treatment for cancer with less adverse side effects.

## 1. Introduction

Epirubicin (EPI) is a chemotherapy anthracycline drug that is less cardiotoxic and more effective than its widely used derivative, doxorubicin [1]; hence, it is recommended as a suitable alternative to doxorubicin [2]. To date, it has been proven to be effective to treat lung, liver, and breast cancer [3,4,5,6,7]. EPI forms a complex with DNA and eventually inhibits the synthesis of macromolecules [8]. Moreover, additional studies reported that EPI acts by the generation of free radicals, which leads to DNA damage or lipid peroxidation. It was also suggested that EPI affects the cell membrane by inhibiting topoisomerase II directly, which induces apoptosis [4,9]. Currently, the conventional dosage form of EPI is administered as an intravenous solution for cancer therapy. Although the current treatment is effective, it also causes severe side effects such as secondary malignancies, extravasation, and tissue necrosis [4].

The main disadvantage of conventional cancer therapies is nonspecific binding, which leads to an inadequate drug amount being delivered to the tumor tissue while also causing adverse effects on normal cells. Due to their unique properties, nanoparticles (NPs) are being used to deliver chemotherapies to cancer cells [10]. Their small size range and large surface area allow them to interact with biological molecules and penetrate cancer cells to deliver drugs and other materials. Furthermore, the main feature of NPs is their capacity to target specific tumor tissues and release their content in a specific fashion to achieve the maximum therapeutic effect with minimum side effects [11,12,13,14,15]. In addition, NPs are efficient at delivering drugs with low solubility due to their ability of encapsulating both hydrophilic and hydrophobic drugs [16,17,18,19]. Nanomaterials such as polymeric NPs, hybrid NPs, gold NPs, liposomes, and quantum dots have proved to be effective diagnostic and therapeutic tools in cancer treatments [20,21,22,23,24].

Furthermore, the exceptional properties of nano-based drug delivery systems (DDSs) enable them to be formulated in different dosage forms requiring various stability properties [25,26,27,28,29,30,31]. Even though nano-based DDSs have various advantages, it is challenging to manufacture them due to their stability issues. The NPs’ stability is an important factor in determining the safety and efficacy of nano-based DDSs. The particles’ size and distribution play an important role in DDSs [32], especially in intravenous formulations. For instance, NPs’ agglomeration can lead to capillary blockage and may cause serious issues in pulmonary DDSs. Hence, monitoring the stability of synthesized DDSs is vital during the pharmaceutical production stages [33,34].

The synthesis of anti-cancer DDSs is attracting the attention of many scientists; EPI, specifically, has been encapsulated in marine carbohydrate as a kind of pH-triggered DDS [35]. Epirubicin has also been formulated as polymeric micelles in the context of sonodynamic therapy [36]. Moreover, treatment with EPI loaded in poly(butyl cyanoacrylate) NPs showed an apoptotic effect on cervical cancer cells [37]. In addition, EPI has been successfully attached to supermagnetic iron oxide to treat skin cancer [38] and has also been combined with silver and gold NPs to synthesize DDS [39,40]. In this study, a biocompatible DDS of EPI was prepared using tri-block poly (lactide) poly (ethylene glycol) poly(lactide) (PLA–PEG–PLA) polymer. This is the first study that combines the PLA–PEG–PLA triblock polymer with epirubicin in an aim to create a highly effective biocompatible and biodegradable DDS that offers sustained release EPI-NPs to treat breast cancer. The prepared EPI-NPs were characterized by different techniques and the therapeutic efficiency was evaluated by performing in vitro studies on estrogen positive breast cancer cells (MCF-7).

## 2. Materials and Methods 

### 2.1. Materials

For nanoparticles’ synthesis, polylactide-block-poly (ethylene glycol)-block-polylactide (Tri-Block- PLA Mn 1500, PEG Mn 900), poly (vinyl alcohol) (PVA) (molecular weight: 89,000–98,000 Da), and chloroform (99.0–99.4% (GC)) were purchased from Sigma–Aldrich Chemical Co. (St. Louis, MO, USA). The drug EPI hydrochloride (HCl) (molecular weight: 579.98 Da) (Alfa Aesar, Ward Hill, MA, USA), was dissolved in dimethyl sulfoxide (DMSO); ACS (UFC Biotech, RUH, KSA). Ultrasonication was performed using an ultrasonic processor (GEX 130; Sonics and Materials Inc., Newtown, CT, USA).

MCF-7 (ATCC HTB-22) breast cancer cells were purchased from the American Type Culture Collection (Manassas, VA, USA) and cultured in Dulbecco’s Modified Eagle Medium (DMEM) 1× + GlutaMax™ containing 10% fetal bovine serum (FBS) and 1% penicillin–streptomycin, then harvested with stable trypsin replacement enzyme (TrypLE™ Express (1×)); all were purchased from (Thermo Fisher Scientific, Waltham, MA, USA). Cells were incubated at 37 °C in a 5% CO_2_ humidified incubator (NU-4750, NuAire, Plymouth, MN, USA). For flow cytometry, an Annexin V-FITC apoptosis detection kit (BMS500FI-100|) was purchased from Invitrogen (Carlsbad, CA, USA). For the stability study, the solutions used included potassium hydroxide (KOH) (Sigma–Aldrich Chemical Co.), Tris HCl (≥99.0% UltraPure; UFC Biotech), and phosphate buffer saline (PBS) (pH: 7.4, 1×) (Thermo Fisher Scientific). The solutions’ pH were measured with the Seven Compact S220 pH meter (Mettler Toledo, Greifensee, Switzerland). All other solutions and chemicals were of analytical grade.

### 2.2. Preparation of EPI Polymeric NPs

The NPs were prepared using a double emulsion method. An amount of 40 mg of PLA–PEG–PLA polymer was dissolved in 2 mL of chloroform and 100 µM of EPI dissolved in DMSO was subsequently added. To create the first emulsion, the sample was placed in an ice bath and ultrasonicated for five minutes (50 s on, 10 s off) at 65% amplitude. Then, 3 mL of 1.5% PVA (prepared in advance by dissolving PVA powder in dH_2_O) was added slowly to the solution and the sample was ultrasonicated using the same previous settings to create the double emulsion. The final formed nanosuspension was stirred for one hour at room temperature (RT) under a fume hood to facilitate the complete evaporation of chloroform. The sample was then centrifuged (Hermle Z 36 HK; HERMLE Labortechnik GmbHk, Wehingen, Germany) in an Eppendorf tube at 14,000 rpm for one hour at RT. The precipitated NPs were next washed with 2 mL of distilled water and recentrifuged for 30 min at the same settings. After collecting the supernatant, the NPs were air-dried overnight under a fume hood. For the control, void NPs were prepared by adding free DMSO instead of the drug using the same exact method.

### 2.3. Characterization of the EPI-Loaded NPs Formulations

#### 2.3.1. Particle Size and Polydispersity Index Analysis

The average particle size and polydispersity index (PdI) were determined by dynamic light scattering (DLS) using a particle size analyzer (ZetaPALS; Brookhaven Instruments, Holtsville, NY, USA) with an angle of detection of 90°. Both the particle size and PdI were measured five times consecutively. The average of the five instrument runs for each was calculated. The statistical analysis of particle size and polydispersity index were performed through Particle Solutions Software-Brookhaven Instruments, NY, USA.

#### 2.3.2. Measurement of Zeta-Potential

Using the aforementioned particle size analyzer (ZetaPALS; Brookhaven Instruments, NY, USA), the zeta-potential of the EPI-NPs was measured by enacting the laser Doppler velocimetry mode. The statistical analysis of zeta potential was performed through Particle Solutions Software-Brookhaven Instruments, NY, USA.

#### 2.3.3. Transmission Electron Microscopy (TEM)

TEM images were collected using a JEM-1400 electron microscope (JEOL, Tokyo, Japan) operating at an acceleration voltage of 120 kV. A drop of the sample (1 mg/mL) was placed on a 400-mesh, carbon-coated copper grid. The samples were air-dried at RT prior to recording measurements.

#### 2.3.4. Measurement of Drug Entrapment Efficiency (%EE) and Drug Release Study

The supernatant collected after the centrifugation processes (mentioned in Section 2.2), was measured using ultraviolet spectrophotometry to determine the amount of excess EPI. The concentration of EPI entrapped in the NPs was measured from the precipitated NPs by dissolving a known amount of the NPs in DMSO to release the drug, then determining the absorbance. The %EE was calculated according to the following equation:

%EE = (amount of entrapped drug)/ (amount of the initially added drug) × 100(1)

For the release study, 4 mg of the EPI-NPs resuspended in 1.5 mL pbs inserted into a dialysis tube (W 25 mm) made from cellulose membrane, Mwt cut off = 14,000 (Sigma–Aldrich, Co., MO, USA). The sample-containing tube was immersed in 14 mL of PBS in a small dark glass bottle containing magnet. The bottle was then closed and placed on a magnetic stirrer at a speed of 4 rpm and temperature of 37 °C. To determine the amount of the released EPI, UV absorbance was measured; each time, 1 mL of the sample was taken for measurement and replaced by PBS to maintain sink conditions. The data were obtained through SoftMax Pro Software-Molecular Devices, CA, USA.

### 2.4. Stability Study

At this stage, 200 µL of void NPs and 200 µL of EPI-NPs were dispersed in 800 µL of the following five different solutions for analysis: ultra-pure water (pH: 7.02), PBS (pH: 7.15), DMEM media with FBS and penicillin–streptomycin (pH: 7.10), HCl (pH: 3.26), and KOH (pH 14.05). The final concentrations of the void NP and EPI-NP dispersions were 2.2 mg/mL and 7.671 μM. The NP dispersions were stored in sealed Eppendorf tubes in the dark at RT. The stability of the NPs was tested daily in all solutions for a duration of 30 days and using the particle size analyzer (ZetaPALS; Brookhaven Instruments, NY, USA), the average of the five instrument runs for each parameter (particle size, PDI and zeta potential) was calculated.

### 2.5. Differential Scanning Calorimetry

The thermodynamic properties of EPI-NPs were studied by differential scanning calorimetry (DSC 412 Polyma; NETZSCH, Selb, Germany) to identify the purity degree of EPI HCl and the level of epirubicin–copolymer interaction. The DSC system was calibrated using the indium calibration standard. Then, a small amount (5–7 mg) of pure EPI, EPI-NPs, and void NPs were weighed in the DSC aluminum pans separately to be analyzed in three separate runs. The starting temperature was 30 °C and was gradually increased up to 250 °C at a rate of 10 °C per minute using nitrogen as a purging gas at a flow rate of 40 mL/min. 

### 2.6. Anticancer Activity of EPI-NPs

#### 2.6.1. Assessment of Anticancer Activity Using Flow Cytometry

An annexin V-FITC apoptosis staining was used to evaluate cell viability as per the manufacturer’s recommendations. Briefly, MCF-7 cells at passage number 12 were seeded (0.4 million) in a T25 culture flask in 3 mL of 10% FBS DMEM complete medium. After 90 min of incubation, cells were treated with EPI (6 nM, 12 nM, 24 nM, and 48 nM) of EPI-NPs. As a control, the cells were also treated with EPI in its free form at the same concentrations. In addition, two T25 flasks containing binding buffer and annexin-V were used as controls. After 90 min of treatment, supernatant and attached cells were collected by centrifugation. The collected cells were washed with PBS, then centrifuged (600× *g*, 5 min, RT); this step was repeated twice. After that, 5 μL of annexin V-FITC was added to the cell suspension; subsequently, cells were incubated for 10 min at RT and then washed with binding buffer. Next, 10 μL of propidium iodide (20 μg/mL) was added to the cells’ suspension and the cell viability was determined by fluorescence-activated cell sorting (FACS), performed using the FACS Canto II flow cytometry system (BD Biosciences, San Jose, CA, USA).

#### 2.6.2. Fluorescence High-Content Imaging

MCF-7 cells were plated in 96-well plates at a density of 10,000 cells per well in an appropriate amount of DMEM media. Cells were treated with free EPI (5 µM, 10 µM, and 15 µM), EPI-loaded NPs (5 µM, 10 µM, and 15 µM), and void NPs for zero, 24, 48, 72, and 96 h. The cells were incubated at 37 °C under the condition of 5% CO_2_. Prior to microscopy, cells were stained with calcein acetoxymethyl (2 µg/mL), HOECHST33342 (5 µg/mL), and propidium iodide (2.5 µg/mL) for 20 min at 37 °C and 5% CO_2_. Then, cells were imaged using the ImageXpress^®^ Micro system and analyzed with the MetaXpress^®^ software (both Molecular Devices, Downingtown, PA, USA). Nuclei were counted in each well and the average fluorescence intensity was calculated. All experiments were performed in triplicate and their outcomes were averaged; resultant values were reported as the mean ± standard deviation.

#### 2.6.3. Statistical Analysis

Data for the stability study were expressed as the mean of five independent experiments ± standard deviation (SD). Linear regression analysis was performed to describe the relationships between a set of independent variables and the dependent variable. Ordinary least squares (OLS) regression tests were performed to compare two groups of quantitative variables: days (X) and zeta potential (Y) and days (X) and particle size (Y). The significance of the results was at the level of *p*-value < 0.05. Statistical operations and calculations were performed using Microsoft^®^ Excel^®^ 2016.

## 3. Results and Discussion

**Figure 1:**
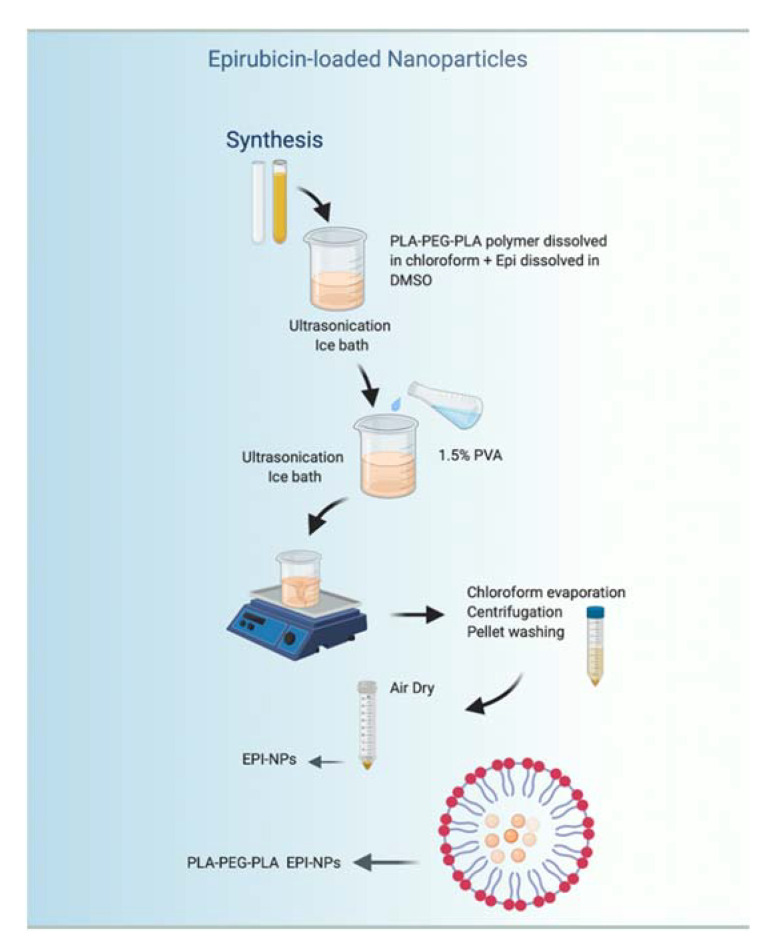
Schematic presentation representing the double emulsion method used to synthesize the epirubicin-loaded polymeric nanoparticles (EPI-NPs). PLA–PEG–PLA: poly (lactide) poly (ethylene glycol) poly(lactide); DMSO: dimethyl sulfoxide; PVA: poly (vinyl alcohol).

**Figure 2:**
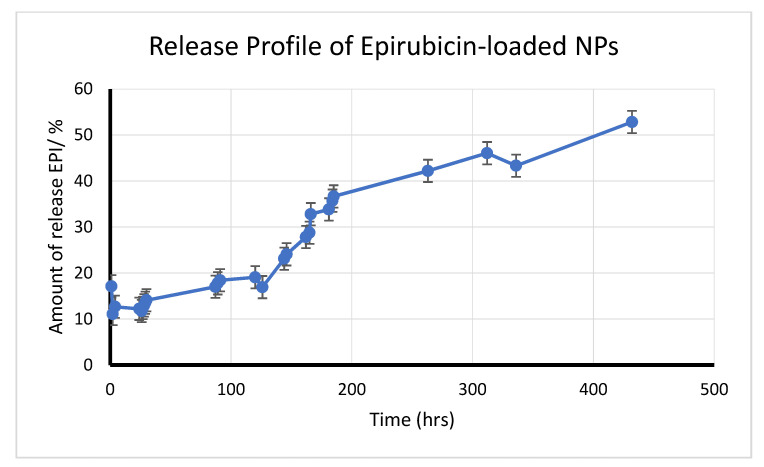
In vitro epirubicin release profile from EPI-NPs over a period of 432 h at 37 °C. The data represent the mean average of three measurements ± SD.

Here, the synthesis of EPI-NPs is described. The EPI-NPs were prepared using a previously reported method [19,41]. The double emulsion method allows for the encapsulation of hydrophobic and hydrophilic drugs [42]. In addition, the double emulsion method provides the nanoparticles with the controlled release feature, which makes them suitable candidates as sustained release drug delivery systems [43,44]. The PLA–PEG–PLA amphiphilic polymer was dissolved in chloroform, and the EPI was dissolved in DMSO; both solutions were ultra-sonicated to form the first emulsion. The ultrasonication breaks down the polymer which results in the formation of self-assembled polymeric micelles. The poly lactic acid (PLA) block forms the central hydrophobic core, while the poly ethylene glycol (PEG) block forms the hydrophilic outer layer of the EPI-NPs [42,43]. Subsequently, PVA was added as an emulsifying agent to form the second emulsion. Figure 1 demonstrates the synthesis method of the EPI-NPs. Furthermore, the drug release was monitored over a period of 432 h (Figure 2). The EPI release is sustained over the whole period of the study. The release profile of EPI shows a sharp release at the beginning followed by a steady sustained release (Figure 2). The release of the EPI in its free form (Supplementary Information), shows the burst release of the EPI within few hours. The results obtained from the drug release assay show the advantage of the PLA–PEG–PLA EPI-NPs due to their sustained release properties. The PLA–PEG polymer-based NPs provide sustained release for different types of active ingredients, such as growth hormones, and other hydrophilic and hydrophobic drugs [41,42,44,45].

### 3.1. Dynamic Light Scattering

**Figure 3:**
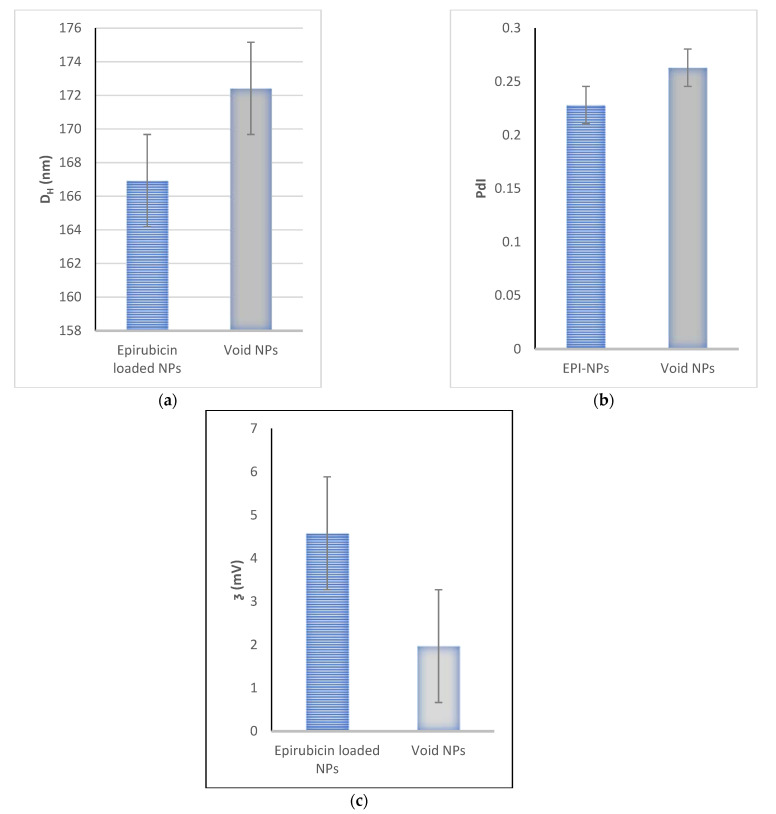
Mean (**a**) particle diameter, (**b**) polydispersity index and (**c**) zeta-potential for epirubicin-loaded NPs and void NPs. The data represent the mean average of the five instrument runs for each sample.

The EPI-NPs were synthesized successfully via the previously described double emulsion method [46]. Figure 3 shows the particle sizes of the EPI-NPs and void NPs, respectively; the EPI-NPs had a mean size of around 166 nm, while the void NPs measured 172 nm. This difference in particle size might be due to the loading of EPI, and its presence in the environment during the synthesis. Furthermore, the synthesized EPI-NPs had a PdI of 0.23, which suggests uniform monodisperse NPs were present. Additionally, the ƺ of the EPI-NPs was 4.58 mV, while that of the void NPs was 1.97 mV (Figure 3c) [32]. The NPs with a zeta potential within the range of −10 and +10 mV are deemed to be neutral [47], therefore the synthesized EPI-NPs will be stable at physiological pH values.

### 3.2. Transmission Electron Microscopy

**Figure 4:**
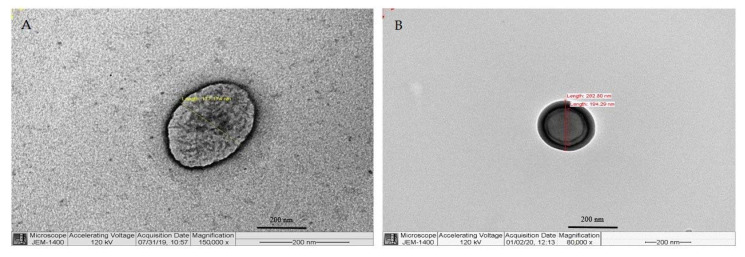
Transmission electron microscopy images showing the morphology of epirubicin-loaded NPs; (**A**) EPI-NPs micrograph, magnification 150,000×. (**B**) EPI-NPs micrograph, magnification 80,000×.

TEM was used to determine the morphology of the synthesized EPI-NPs. Figure 4 includes images of the EPI-NPs, where spherical, homogeneous NPs with no aggregation are clearly visible. The particle size obtained by TEM was similar to that revealed by DLS. Figure 4B shows a fine membrane surrounding the EPI-NPs; this polymeric membrane represents a protective feature to support the delivery of the active ingredient (EPI) to the target site.

### 3.3. Entrapment Efficiency

In this study, the synthesis of the EPI-NPs was optimized to achieve a high encapsulation efficiency, thus improving the biopharmaceutical properties, to ensure an enhanced efficacy of EPI. The encapsulation efficiency was obtained by measuring EPI UV absorbance (for both the encapsulated drug and the excess in the supernatant); the %EE was 82% from both measurements. The EE mainly depends on the polymer composition, drug solubility and functional groups. The use of PVA as a surfactant maintains the stability of the emulsion, specifically during solvent evaporation. Some studies have shown that the use of PLA–PEG copolymers and its end-group derivative nanoparticles has the advantage of increasing drug loading and entrapment efficiency. This can be obtained by adjusting the PEG/PLA ratio to increase the efficiency of hydrophobic drugs [44,48]. Furthermore, the emulsifier plays a key role in the EE [19]. For instance, in an attempt to encapsulate EPI, Chang et al. modified the emulsifying agent and the pH values of the polymerization medium to increase the entrapment efficiency of the EPI [49]. In the same context, Esim et al. encapsulated EPI within poly D,L-lactic-*co*-glycolic acid (PLGA) and they used several surfactants to increase the encapsulation of EPI [50].

### 3.4. Stability Study

The stability and aggregation behavior of the EPI-NPs and void NPs in different solutions were evaluated. Measurements were taken at different time points over a duration of 31 days. This study evaluates the influence of pH and different solutions on the stability of the EPI-NPs. The stability of the EPI-NPs was evaluated in different pH solutions. The different pH solutions used in this study covered the different environments the NPs might encounter if used in vivo. The pH ranged from acidic, physiological pH and basic pH values; the information reported from this study will determine how to best store EPI-NPs to maintain maximum efficacy [28,44].

The surface charges of the NP suspension were assessed by measuring the ƺ (Table 1 and Table 2). Generally, the results of EPI-loaded NP suspensions showed a steady trend, with similar ƺ averages −2.7 mV and 0.21 mV for days 0 and 31 consecutively, although the KOH suspension exhibited high fluctuations among the readings. Moreover, the EPI-NPs in HCl suspension showed a slight decrease in positive charges. On the other hand, the results for the void NP suspensions showed notable changes in ƺ with variations in measurements; specifically, the ƺ of both the HCl and PBS NPs suspensions shifted towards positive charges (day 0: −0.26 mV vs. day 31: −0.09 mV for the HCl suspension and day 0: 0.150 mV vs. day 31: −0.5 mV for the PBS suspension) in contrast with that of NPs suspended in DMEM media, which showed a trend toward positive charges (day 0: −0.37 mV vs. day 31: −0.16 mV). The ƺ of the NP suspended in HCl and water showed no significant changes.

**Figure 5:**
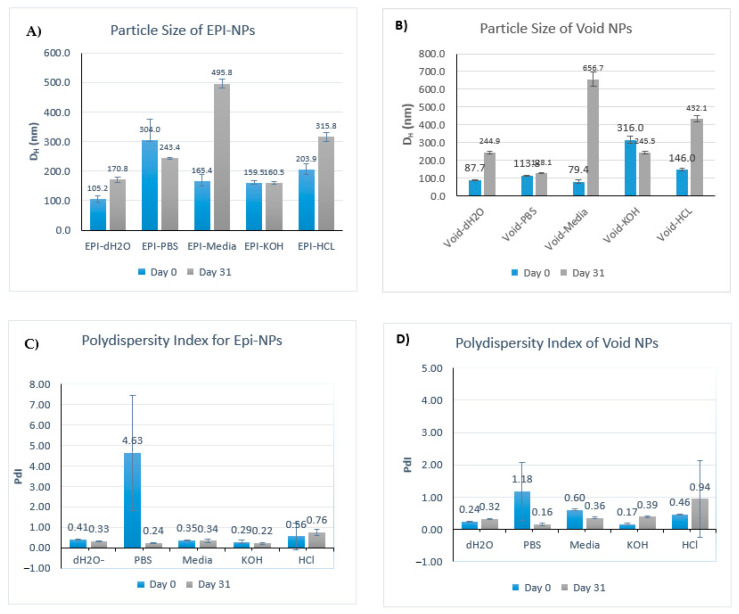
(**A**) Mean particle diameter at day 0 and day 31 for epiribucin-loaded NPs, (**B**) mean particle diameter at day 0 and day 31 for void NPs, (**C**) mean polydispersity index (PdI) at day 0 and day 31 for epiribucin-loaded NPs and (**D**) mean polydispersity index (PdI) at day 0 and day 31 void NPs. The data represent the mean average of the five instrument runs for each sample ± SD.

Regarding the particle size (Figure 5A,B and Table 3), the EPI-loaded and void NPs showed similar trends. For both EPI-NPs and void NPs suspended in water and HCl, the average particle size increased significantly. EPI-loaded and void NPs immersed in PBS showed a slight increase in the average particle diameter (193 nm when freshly prepared). For the NP KOH suspension, no significant change in particle size was observed even after 31 days of preparation. Meanwhile, the average diameter of both void and EPI-NPs immersed in DMEM media increased significantly to become 495 nm after 31 days.

In regard to the polydispersity index, PdI, (Figure 5C,D and Table 4) EPI-loaded NPs immersed in water, DMEM media, and KOH showed no significant change, with results being slightly polydispersed. The PdI for EPI-loaded NPs immersed in HCl showed a small increase (Day 0: 0.56 vs. Day 31: 0.76). In contrast with EPI-loaded NPs in PBS suspension, NPs showed a very high PdI index (4.63) immediately after their addition; however, the PdI decreased significantly (0.24) and was within the monodisperse range by the end of the study.

For the void NPs, the PdI of NPs in KOH and HCl suspensions indicated polydispersity and significantly increased during the study (day 0: 0.17 vs. day 31: 0.39 for KOH; day 0: 0.56 vs. day 31: 0.76 for HCl). For NPs in the water suspension, a slight shift toward the polydisperse range (day 0: 0.24 vs. day 31: 0.32) was observed, while, for NPs in DMEM media suspension, a shift toward the monodisperse range was observed (day 0: 0.60 vs. day 31: 0.36). In a similar way to EPI-loaded NPs, after the initial addition of void NPs to PBS, the PdI indicated high polydispersity (1.18) that decreased (0.16) over the course of the study. The PdI results confirm that stable monodispersed NPs remained monodispersed and non-aggregated even after 30 days of synthesis.

**Figure 6:**
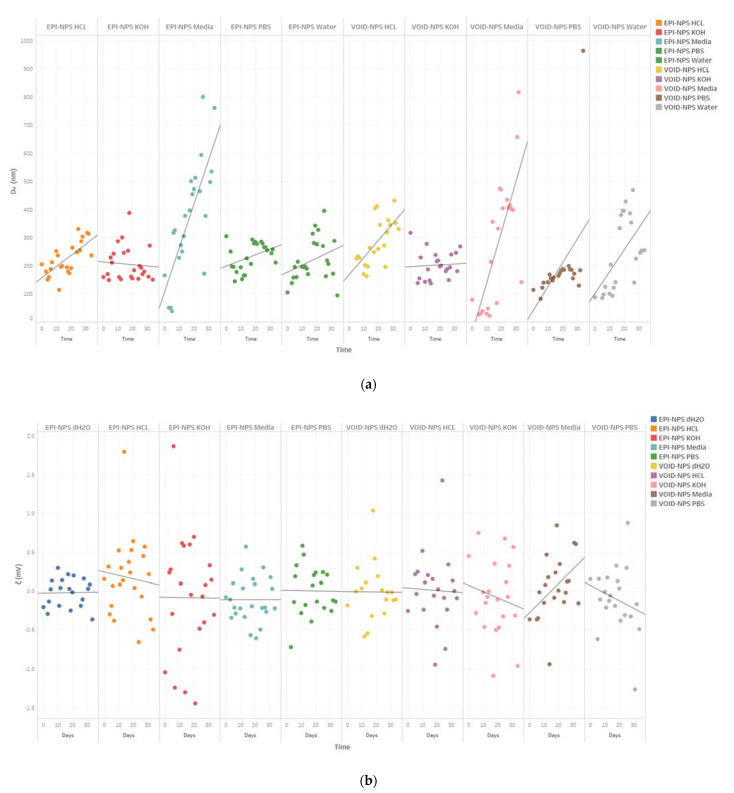
(**a**) Timepoint particle size measurements for EPI-NPs and void-NPs for a period of 30 days, (**b**) timepoint measurements of zeta potential measurements for EPI-NPs and void NPs over a period of 30 days.

**Figure 7:**
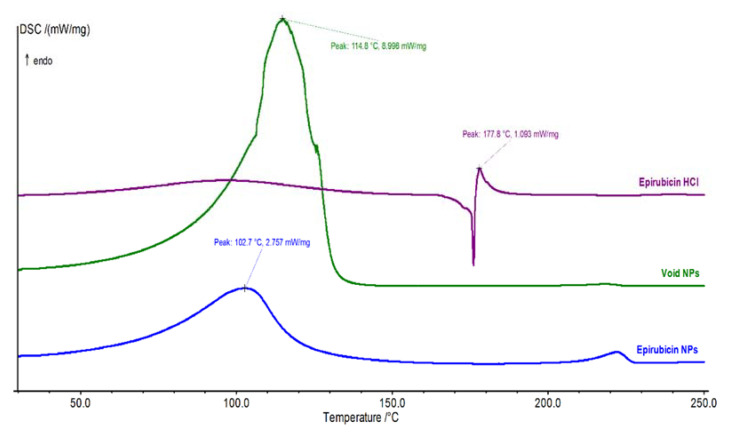
Differential scanning calorimetry thermogram of epirubicin, void and epirubicin-loaded NPs.

The DLS results indicated that the NPs were sufficiently stable during 31 days of storage in the dark at RT (Figure 6). In general, EPI-NPs have a better stability profile than void NPs; Figure 7 below shows that the EPI-NPs are stable for the period of 30 days at physiological pH values. The particle size and surface charge of EPI-NPs remained within the acceptable range for a period of 30 days. 

The increase in size of the EPI-NPs immersed in DMEM media can be attributed to the adsorption of FBS found in the media. Following the cross-linkage of polymeric NPs with bovine serum albumin (BSA), Palanikumar et al. analyzed the 26 most abundant serum proteins and found that PLGA NPs had higher adsorption than the BSA-PLGA NPs did [51]. However, the increase in size observed could be attributed to other reasons such as the presence of the hydrophilic PEG layer with low surface charges, which may reduce the serum protein absorption [41]. The increase in the average diameter of particles in HCl is combined with an increase in polydispersity. The acidic environment might lead to an aggregation of particles, causing the hydrolysis of the ester bonds in the polymer chain, which leads to the degradation of the particle cores [51]. A study by Lazzari et al. in 2012 showed that polymeric NPs do aggregate in gastric juice [52]. 

In addition to the pH values, many factors can cause NPs aggregation, including salts and enzymes [52]. This may explain the immediate aggregation of NPs when added to PBS and media. The variations found in ƺ measurements in void NPs suspensions in PBS and media and the EPI-NPs’ suspension in HCl could be attributed to the same reasons. In conclusion, from the above results, it can be determined that the EPI-NPs are most stable at physiological pH values, which makes them suitable to be formulated in different dosage forms without restrictions. Figure 6 below represents timepoint particle size and zeta potential measurements for EPI-NPs and void-NPs over a period of 30 days.

### 3.5. Differential Scanning Calorimetry

The thermodynamic properties of EPI-NPs were studied by differential scanning calorimetry (DSC 412 Polyma; NETZSCH, Selb, Germany) to identify the purity degree of EPI HCl and the level of epirubicin–copolymer interaction. The DSC system was calibrated using the indium calibration standard. Then, a small amount (5–7 mg) of pure EPI HCl, EPI-NPs, and void NPs were weighed in the DSC aluminum pans separately to be analyzed in three separate runs. The starting temperature was 30 °C and was gradually increased up to 250 °C at a rate of 10 °C per minute using nitrogen as a purging gas at a flow rate of 40 mL/min. The DSC thermograms are presented in Figure 7.

Figure 7 shows the thermograms of pure EPI, EPI-NPs, and void NPs. The SDC thermogram of pure EPI elicited an endothermic (melting) peak at 184.6 °C, corresponding to its melting point, which is very closed to the labeled value (185 °C) [53,54]. The void and EPI-NPs showed mid-broad endothermic peaks at 114.8 °C and 102.7 °C, respectively, due to the melting of the copolymer (PLA–PEG–PLA) matrix of NP formulations. The difference between the void and EPI-NPs is reflected as a little peak shift, where the presence of EPI within the polymeric matrix led to a reduction in its strength of intermolecular interaction force and a decrease in the required transition energy, consecutively [55]. Furthermore, as the melting temperature of the PLA is 130–180 °C, the merging of the PEG inside the PLA matrix in a form of PLA–PEG–PLA triblock copolymer (66.7% PLA) leads to a reduction in its strength and a decrease in the melting temperature below 115 °C [56]. This explains the appearance of the EPI HCl and void NPs peaks at 102.7 and 114.8 °C, respectively.

The EPI-NPs’ thermogram presented in Figure 7 shows no endothermic peak in the area of 180 °C to 190 °C, which indicates the collapse of EPI’s crystalline structure and turns into the molecular level, allowing the EPI to interact with the polymeric matrix system, and reinforce its thermal stability as well. This theory is supported in a previous study by Lavor et al. [57]. Additionally, this theory is supported by the appearance and shifting of the small endothermic peak of the pure EPI HCl from 184.6 °C to 222.1 °C, indicating the high stability of EPI HCl within the polymeric NP system. 

### 3.6. The Effect of EPI-NPs on Estrogen Positive Cancer Cells MCF-7

**Figure 8:**
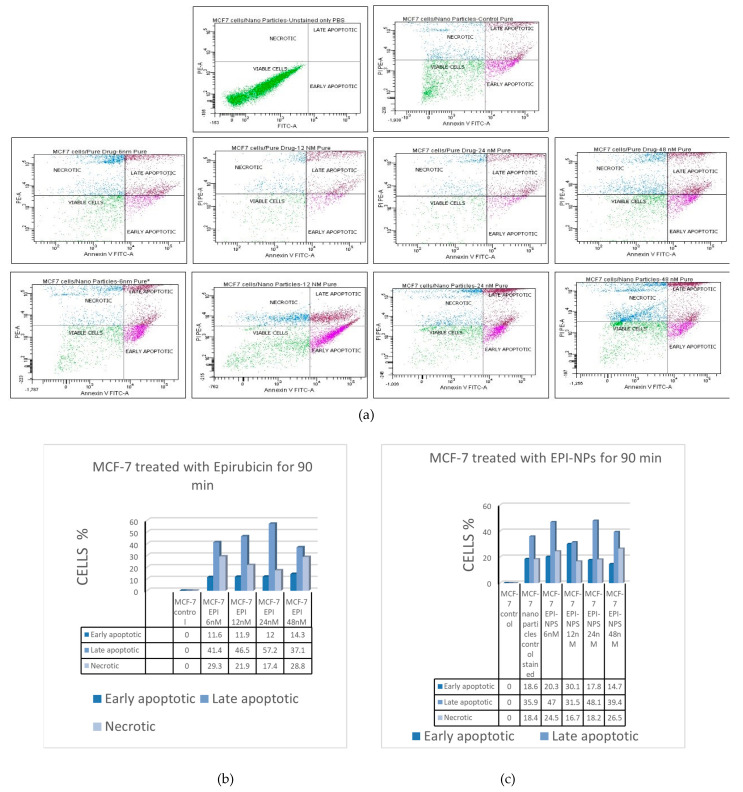
(**a**) Flow cytometry results for MCF-7 cells, incubated with 6 nM, 12 nM, 24 nM and 48 nM EPI and 6 nM, 12 nM, 24 nM and 48 nM EPI-NPS (**b**) column graph representing the percentage of early apoptotic, late apoptotic, and necrotic MCF-7 cells incubated with 6 nM, 12 nM, 24 nM and 48 nM EPI and (**c**) column graph representing the percentage of early apoptotic cells, late apoptotic cells, and necrotic MCF-7 cells incubated with 6 nM, 12 nM, 24 nM and 48 nM EPI-NPs.

Flow cytometry has been used in this study to evaluate the effect of EPI-NPs on the MCF-7 cell line and to compare it with the effect of free-form EPI on the same cells (Figure 8). The MCF-7 cells were treated with 6 nM, 12 nM, 24 nM, and 48 nM of EPI-NPs and 6 nM, 12 nM, 24 nM, and 48 nM EPI for 90 min. Figure 6A–C show the effect of EPI on the MCF-7 cells; notably, early apoptosis, late apoptosis, and necrosis were observed within the 90-min treatment period. Similarly, in the presence of EPI-NPs, the MCF-7 cells showed a similar apoptotic profile as that triggered by the free-form EPI. In conclusion, the synthesized EPI nanocarriers showed more apoptosis toward the breast cancer cells, which suggests greater cytotoxicity is correlated with the EPI-NPs.

Flow cytometry was used in this study to evaluate the effect of EPI-NPs on the MCF-7 cell line and to compare it with the effect of free-form EPI on the same cells. The MCF-7 cells were treated with 6 nM, 12 nM, 24 nM, and 48 nM of EPI-NPs and 6 nM, 12 nM, 24 nM, and 48 nM EPI for 90 min. Figure 8A–C show the effect of EPI on the MCF-7 cells; notably, early apoptosis, late apoptosis, and necrosis were observed within the 90-min treatment period. Similarly, in the presence of EPI-NPs, the MCF-7 cells showed a similar apoptotic profile as that triggered by the free-form EPI. In conclusion, the synthesized EPI nanocarriers showed the synthesized EPI nanocarriers showed more apoptosis toward the breast cancer cells, which suggests greater cytotoxicity is correlated with the EPI-NPs.

### 3.7. Fluorescence Imaging of MCF-7 Cells Treated with EPI-NPS

**Figure 9:**
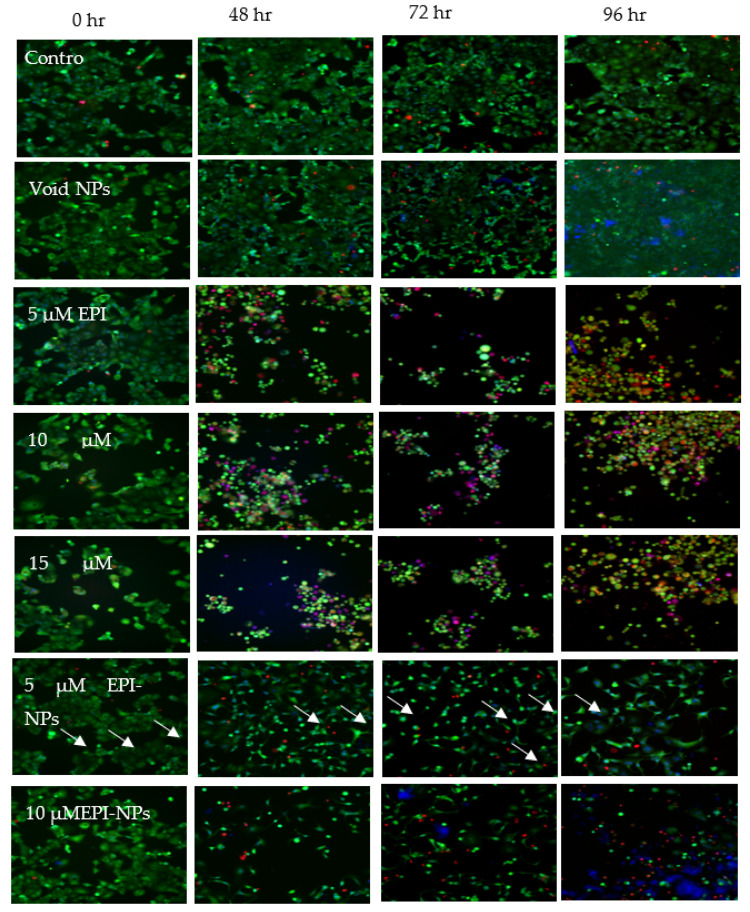
Fluorescence imaging of MCF-7 cells. MCF-7 cells treated with epirubicin (5 µM, 10 µM and 15 µM), epirubicin-loaded nanoparticles (5 µM, 10 µM and 15 µM) and void nanoparticles for 0, 24, 48, 72, 96 h. After treatment, cells were stained with calcein (orange) AM (2 μg/mL), HOECHST33342 (blue) (5 μg/mL), and propidium iodide (red) (2.5 μg/mL) for 20 min. Scale bar of all images is 50 µm. The white arrows points at the condensed nuclei (blue) in apoptotic cells. The red stained cells indicate cell death detected by the propidium iodide staining. The MCF-7 cells show more apoptosis when incubated with 5 µM EPI-NPs and 10 µM EPI-NPs for 96 h, compared with the control and void NPs.

Different concentrations of EPI-NPs were incubated with MCF-7 cells to identify necrotic cells by staining with HOECHST33342/PI staining. Figure 9 shows the images of the MCF-7 cells after treatment at different time points (0, 48, 72, and 96 h). It is clear that the EPI-NPs achieved more apoptosis at 96 h compared with the control; this is believed to be due the sustained release properties of the PLA–PEG–PLA polymer used to synthesize the EPI-NPs.

**Figure 10:**
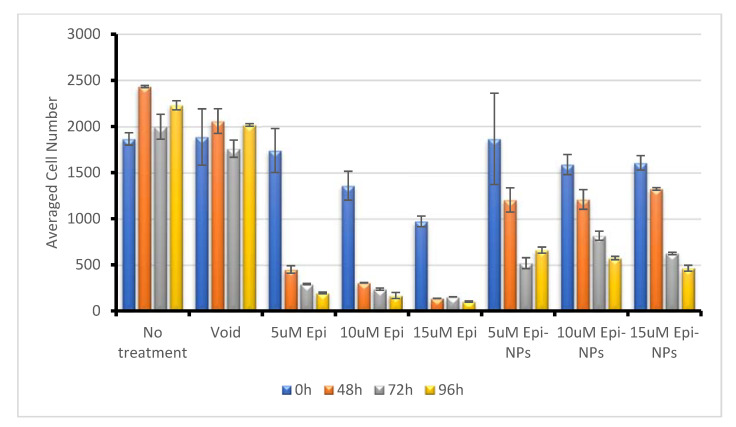
Average cell number for MCF-7 cells treated with epirubicin (5 µM, 10 µM and 15 µM), epirubicin-loaded nanoparticles (5 µM, 10 µM and 15 µM) and void nanoparticles for 0, 24, 48, 72, 96 h.

The high-content imaging experiment showed that treatment with EPI-NPs causes cell death as compared with the control and void-NPs treatments. Figure 9 shows that the cells tend to condense and decrease in number, especially when incubated for longer durations. The observed cell condensation indicates that apoptosis is occurring among the cells; the dye used to stain the cells, HOECHST, binds to the cellular DNA, therefore it reveals the nuclear condensation when cells undergo apoptosis [58,59,60,61]. Moreover, Figure 10 shows a time- and dose-dependent cellular response. At zero hours, the cell count was not affected by the added EPI-NPs, while, on the other hand, at 96 h, the number of cells had dramatically decreased, especially after being dosed with higher concentrations of EPI-NPs. The EPI-NPs are encapsulated with the PLA–PEG–PLA triblock polymer, which gives these NPs the characteristic of sustained release.

## 4. Conclusions

EPI-NPs were successfully synthesized and optimized. The EPI-NPs were characterized by different methods. They possess a relatively small particle size, which allows them to be internalized and delivered in cancerous tissue. With the optimized method of synthesis, the EPI-NPs had an encapsulation efficiency of around 82%, which proves the efficient synthesis method used for the preparation. The flow cytometry studies showed that the EPI-NPs have an apoptotic effect on MCF-7 breast cancer cells. In addition, the high-content imaging studies revealed a gradual decrease in cancer cells number after treatment with EPI-NPs. The stability of EPI-NPs was intensively investigated to determine their potential use as DDSs. In addition, the EPI-NPs showed a sustained drug release profile, and they were stable at different pH values and in different conditions. However, they were mostly stable as physiological pH values, which makes them prime candidates for different pharmaceutical dosage forms. Further in vivo studies are recommended as a next step to study the pharmacokinetics of the EPI-NPs in animal models.

## Figures and Tables

**Table 1:** Mean zeta potential at day 0 and day 31 for void NPs; the data represent the mean average of the five instrument runs for each sample ±SD. HCL: hydrochloride; KOH: potassium hydroxide; PBS: phosphate buffer saline; DMEM: Dulbecco’s Modified Eagle Medium.

	Mean Zeta Potential (ζ)/ mV for Void NPs	
	Day 0	Day 31
H_2_O (pH 7.02)	−0.18 ± 0.5	−0.11 ± 0.2
PBS (pH 7.15)	0.15 ± 0.4	−0.50 ± 1.5
DMEM Media (pH 7.10)	−0.37 ± 0.04	−0.16 ± 0.1
KOH (pH 14.05)	0.45 ± 0.4	−0.97 ± 1.1
HCL (pH 3.26)	−0.26 ± 0.4	−0.09 ± 0.2

**Table 2:** Mean zeta potential at day 0 and day 31 for epirubicin-loaded NPs. The data represent the mean average of the five instrument runs for each sample ±SD.

	Mean Zeta Potential (ζ)/mV for EPI-NPs	
	Day 0	Day 31
H2O (pH 7.02)	−0.21 ± 0.9	0.03 ± 0.1
PBS (pH 7.15)	−2.56 ± 11.4	−0.26 ± 0.3
DMEM Media (pH 7.10)	−0.08 ± 0.3	0.17 ± 0.5
KOH (pH 14.05)	−1.05 ± 0.4	0.33 ± 0.5
HCL (pH 3.26)	0.16 ± 0.1	0.21 ± 0.7

**Table 3:** Mean particle size at day 0 and day 31 for EPI-NPs and void NPs. The data represent the mean average of the five instrument runs for each sample ±SD.

		Solution									
		Water		PBS		Media		KOH		HCL	
		Day 0	Day 31	Day 0	Day 31	Day 0	Day 31	Day 0	Day 31	Day 0	Day 31
**Particle size**	**EPI-NPs**	105.2 ± 12	170.8 ± 9.4	304.8 ± 72.8	243.4 ± 2.8	165.4 ± 23.3	495.8 ± 15.5	159.5 ± 7.11	160.5 ± 4.5	203.9 ± 19.7	315.8 ± 16
	**Void NPs**	87.7 ± 1.2	244.9 ± 7.9	113.8 ± 2.5	128.1 ± 3	79.4 ± 9.6	656.7 ± 41.5	316.5 ± 21.2	245.5 ± 7.3	146 ± 8	432.1 ± 17.2

**Table 4:** Mean polydispersity at day 0 and day 31 for EPI-NPs and void NPs. The data represent the mean average of the five instrument runs for each sample ±SD.

		Solution									
		Water		PBS		Media		KOH		HCL	
		Day 0	Day 31	Day 0	Day 31	Day 0	Day 31	Day 0	Day 31	Day 0	Day 31
**Polydispersity**	**EPI-NPs**	0.41 ± 0.02	0.33 ± 0.02	4.63 ± 2.8	0.24 ± 0.02	0.35 ± 0.03	± 0.34 ± 0.08	0.29 ± 0.1	0.22 ± 0.04	0.56 ± 0.65	0.76 ± 0.14
	**Void NPs**	0.24 ± 0.02	0.32 ± 0.02	1.18 ± 0.9	0.16 ± 0.04	0.60 ± 0.03	0.36 ± 0.02	0.17 ± 0.03	0.39 ± 0.03	0.46 ± 0.01	0.94 ± 1.2

## Data Availability

The data presented in this study are available in this article or https://www.mdpi.com/article/10.3390/polym13081212/s1.

## Supplementary Materials

The following are available online at https://www.mdpi.com/article/10.3390/polym13081212/s1, Figure S1: In vitro epirubicin release profile.
