# Supplementary figures and images for: Development of Epirubicin-Loaded Biocompatible Polymer PLA–PEG–PLA Nanoparticles: Synthesis, Characterization, Stability, and In Vitro Anticancerous Assessment
Source: Polymers (Basel). 2021 Apr 9;13(8):1212. doi: 10.3390/polym13081212 (PMC8070301; doi:10.3390/polym13081212)

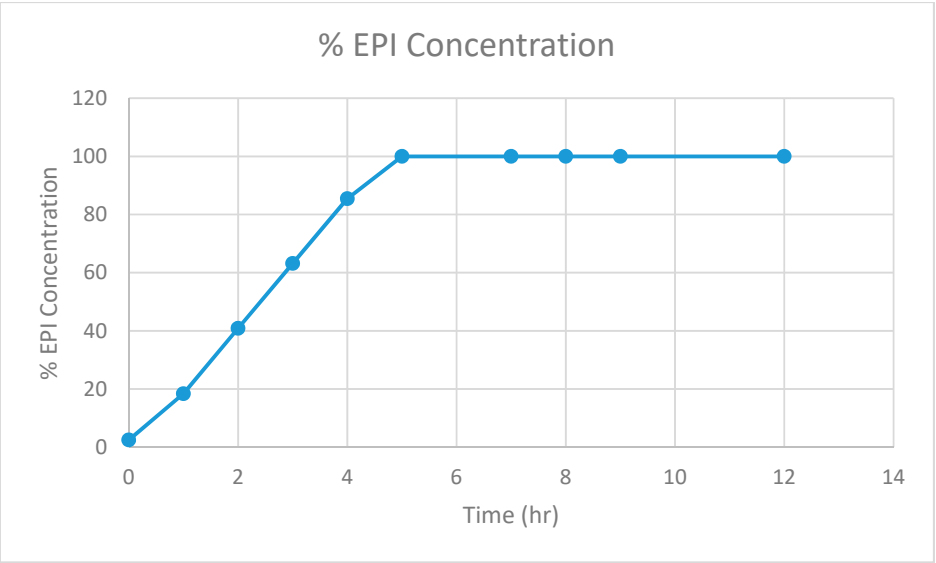

Supplement: Supplementary file 1 [file polymers-13-01212-s001.pdf]
